# Trispecific killer engager 161519 enhances natural killer cell function and provides anti-tumor activity against CD19-positive cancers

**DOI:** 10.20892/j.issn.2095-3941.2020.0399

**Published:** 2020-12-15

**Authors:** Ying Cheng, Xiaodong Zheng, Xuefu Wang, Yongyan Chen, Haiming Wei, Rui Sun, Zhigang Tian, Haoyu Sun

**Affiliations:** 1 Hefei National Laboratory for Physical Sciences at Microscale, The CAS Key Laboratory of Innate Immunity and Chronic Disease, School of Basic Medical Sciences, Division of Life Sciences and Medicine, Institute of Immunology, University of Science and Technology of China, Hefei 230027, China; 2 Research Unit of NK Cell Study, Chinese Academy of Medical Sciences, Beijing 100864, China

**Keywords:** Trispecific antibody, trispecific killer engager (TriKE), NK cell, B-cell lymphoma, tumor immunotherapy

## Abstract

**Objective:**

Natural killer (NK) cells have gained considerable attention due to their potential in treating “cold tumors,” and are therefore considered as one of the new strategies for curing cancer, by using worldwide development of their new possibilities and interventions with NK cell-related therapeutic products.

**Methods:**

We constructed a trispecific killer engager (TriKE) consisting of anti-CD16, IL-15, and anti-CD19. This TriKE was designed to attract CD19^+^ tumor cells to CD16^+^ NK cells, whereas IL-15 sustained the proliferation, development, and survival of NK cells.

**Results:**

Treatment with 161519 TriKE in the presence of CD19^+^ targets upregulated expression of CD69, CD107a, TRAIL, IFN-γ, and TNF-α in NK cells, and significantly improved the proliferation and cytotoxicity of NK cells. NK cells “armed” with 161519 TriKE showed stronger cytolysis against CD19^+^ targets compared with that of “unarmed” NK cells. A preclinical model of B-cell lymphoma in human peripheral blood mononuclear cell-reconstituted xenograft mice showed significant inhibition of tumor growth and prolonged overall survival after treatment with 161519 TriKE, when compared with that in control mice or mice treated with 1619 BiKE. Combined use of IL-2 was a more effective treatment with 1619 BiKE, when compared with that using 161519 TriKE.

**Conclusions:**

The newly generated 161519 TriKE enhanced the proliferation, activation, cytokine secretion, and cytotoxicity of NK cells in the presence of CD19^+^ tumor cells. The 161519 TriKE aided inhibition of tumor growth and prolonged the overall survival of murine xenografts, and could be used to treat CD19-positive cancers.

## Introduction

Bispecific antibodies (BsAbs) represent the second generation of antibody-based immunotherapy. Instead of conventional monoclonal blockade, they provide new therapeutic strategies against tumors by attracting immune cells to transformed cells^1^. Most of the BsAbs in preclinical and clinical investigations have been dominated by bispecific T-cell engagers (BiTEs), which consist of 2 binding sites: One directed at tumor cells and the other directed at CD3^2^. The most representative BiTE is blinatumomab^1^. Very low doses of blinatumomab in non-Hodgkin’s lymphoma patients leads to the elimination of tumor cells in blood, as well as partial and complete tumor regression^3^. Continuous intravenous infusion of blinatumomab has been shown to result in complete remission (CR) or CR with partial hematological recovery of peripheral blood counts (CRh) in 43% of patients with relapsed or refractory (R/R) B-precursor acute lymphoblastic leukemia (ALL)^4,5^. However, despite these promising outcomes, adverse effects [e.g., cytokine release syndrome (CRS) and neurological toxicity] are commonly associated with BiTEs^2^.

Besides T cells, the anti-tumor roles of natural killer (NK) cells have been studied extensively. Reduced numbers and impaired function of NK cells are associated with progression of various types of cancer^6^. Introduction of the term “cold tumor” has led to the emergence of NK cells in anti-tumor immunotherapy. Cold tumors are “immunologically ignorant” and express minimum or no major histocompatibility complex (MHC) class I molecules on their surface^7^, and are barely detected by T cells; however, they can be recognized and targeted by NK cells^8–11^.

Bispecific killer cell engagers (BiKEs) are emerging as an alternative to BiTEs. BiKEs are designed so that one “arm” targets tumor antigens, whereas the other arm binds to the NK cell receptor (CD16). In this way, NK cells and tumor cells are attracted together to form an “immunological synapse,” which results in the sustained stimulation of NK cells and stronger lysis of tumor cells^12,13^. Treatment with CD16 × CD33 BiKE specifically triggers NK-cell cytotoxicity and cytokine release against CD33^+^ targets and refractory acute myeloid leukemia (AML) targets^14^. Treatment with CD16 × CD33 BiKE enhances degranulation and tumor necrosis factor (TNF)-α, and interferon (IFN)-γ production against CD33^+^ myelodysplastic syndrome targets and reverses immunosuppression of NK cells by myeloid-derived suppressive cells^15^. AFM13 is a tetravalent bispecific CD30/CD16A tandem diantibody consisting of 2 binding sites for CD16A and 2 binding sites for CD30. AFM13 represents a promising therapy for Hodgkin’s lymphoma, and is the most rapidly progressing antibody based on NK-cell redirection^16^.

Although treatment with BiKEs can enhance the activation and function of NK cells against tumor cells, they have been limited by the inability of NK cells to survive^1^. To overcome this limitation, incorporation of a modified human interleukin (IL)-15 crosslinker has been used to produce a 161533 trispecific killer cell engager (TriKE). The 161533 TriKE induces superior cytotoxicity, degranulation, and cytokine secretion of NK cells against CD33^+^ targets, and increases the survival and proliferation of NK cells compared with that of 1633 BiKE^17,18^. In addition, 161533 TriKE restores tumor-induced dysfunction and repression of NK cells^17,18^. The 1615133 TriKE targeting CD133^+^ cells have shown greater NK cell-mediated cytotoxicity, expansion of NK cells, and IFN-γ production in NK cells compared with that of 16133 BiKE^19^. CD19-targeting 161519 TriKE, developed by Felices et al.^20^, has demonstrated the potential to drive a potent activating and proliferative signal to NK cells, inducing killing of a CD19-expressing Burkitt’s lymphoma cell line and primary chronic lymphocytic leukemia (CLL) targets. The 161519 TriKE also shows better results in the directed killing of CLL cells *in vitro,* when compared with that of rituximab^20^. A novel NK cell engager targeting the activating receptors, NKp46 and CD16, on NK cells and a tumor antigen on cancer cells has been reported to show higher killing potency than that of any therapeutic antibodies targeting the same tumor antigen^21^.

We constructed a TriKE consisting of anti-CD16, human IL-15, and anti-CD19, similar to that described by Felices et al.^20^. This 161519 TriKE was developed for treatment of CD19-positive cancers and was designed to redirect NK cells *via* their CD16 to kill CD19^+^ target cells; meanwhile, IL-15 aided the development, proliferation, and survival of NK cells. Use of 161519 TriKE significantly improved the interaction between NK cells and CD19^+^ tumor cells *in vitro*, and enhanced the proliferation, activation, cytotoxicity, and cytokine secretion of NK cells. In addition, we analyzed the anti-tumor efficacy of 161519 TriKE using a preclinical model of B-cell lymphoma in human peripheral blood mononuclear cell (PBMC)-reconstituted xenograft mice. Treatment with 161519 TriKE inhibited tumor growth significantly and prolonged the overall survival of tumor-bearing mice compared with that of control mice or mice treated with 1619 BiKE. Treatment with 161519 TriKE combined with IL-2 also showed impressive outcomes. Importantly, combined use of IL-2 was more effective in the treatment of 1619 BiKE, when compared with that observed using 161519 TriKE.

## Materials and methods

### Ethical approval of the study protocol

The study protocol was approved by the Ethics Committee of the University of Science and Technology of China (Approval No. USTCACUC1801012).

### Mice, cell lines, and reagents

Female NOD.Cg-*Prkdc*^scid^*Il2rg^tm1Wjl^* (NOG) mice were kindly provided by Dr. Yangxin Fu from the University of Texas Southwestern Medical Center (Dallas, TX, USA). Mice were kept in specific pathogen-free conditions according to the National Guidelines for Animal Usage in Research (set by the Chinese government) at the University of Science and Technology of China. Mice between 6 weeks and 8 weeks of age were used.

Cell lines (Namalwa, Daudi, Raji, and MM.1S) were purchased from the Cell Bank of the Type Culture Collection of Chinese Academy of Sciences (Shanghai, China). The Karpas 422 cell line was purchased from BNBIO (Beijing, China). The cell lines were cultured at 37 °C in an atmosphere of 5% CO_2_ in RPMI 1640 medium (HyClone, Logan, UT, USA) supplemented with 10% fetal bovine serum, penicillin (100 U/mL) and streptomycin (100 U/mL). All cells were passaged every 2–3 days. Rituximab was purchased from MedChemExpress (Monmouth Junction, NJ, USA) and rituximab (100 nM) was used in the *in vitro* experiments.

### Construction, expression, and purification of 161519 TriKE

The 161519 TriKE was produced using the method of Felices et al.^20^. The 161519 gene fragment encoding the anti-CD16 single-chain variable fragment (scFv)^16^, a linker sequence, PSGQAGAAASESLFVSNHAY, N72D-mutated human IL-15, a linker sequence EASGGPE, and anti-CD19 scFv^22^ were cloned into a pET21d vector. The plasmid was transformed into *Escherichia coli* strain BL21 (DE3). Expression of the hybrid gene was induced by the addition of isopropyl-β-D-thiogalactopyranoside (IPTG) for 2 h. After sonication and centrifugation, cell pellets were extracted with buffer containing Tris (50 mmol/L), NaCl (50 mmol/L), 5% Triton X-100, 0.3% sodium deoxycholate, 10% glycerin, and EDTA (5 mmol/L) adjusted to pH 8.0. Inclusion bodies were washed 4 times. Inclusion bodies were suspended in dissolving buffer [Tris (100 mM), 2.5% sodium N-lauryl sulfate (SLS)], and incubated at room temperature with rapid stirring for 20 h for air-oxygenation of the –SH groups after addition of CuSO_4_ (50 μM) to the solution^16^. The SLS buffer was removed, followed by the addition of 6 M urea and 10% 1-X8 resin (200–400 mesh, chloride form). After incubation for 20 min at room temperature, the resin was removed by filtration. The protein solution was diluted (20-fold) with refolding buffer [Tris (50 mM), L-arginine (0.5 M), EDTA (5 mM), 20% glycerin, pH 8.0], and then incubated for 2 days at 4 °C. Refolded protein was dialyzed against dialysis buffer [Tris-HCl (20 mM), pH 8.0]. SDS-PAGE was conducted using Coomassie Blue Fast Staining Solution (Beyotime, Shanghai, China) to evaluate protein purity. The protein was also quality checked by liquid chromatography-electrospray ionization-time-of-flight mass spectrometry using a system from Sangon Biotech (Shanghai, China).

### Isolation and purification of NK cells and T cells

Blood samples were collected from adults at the Blood Center of Anhui Province (Hefei, China). PBMCs were isolated by centrifugation through a density gradient according to the manufacturer’s instructions (GE Healthcare, Chicago, IL, USA). Negative selection was conducted to purify NK cells and T cells using a MACS kit according to the manufacturer’s instructions (Miltenyi Biotec, Bergisch Gladbach, Germany).

### *In vitro* cell culturing system

For the binding assay, tumor cells were incubated with various concentrations of 161519 TriKE for 30 min at 4 °C. After washing, tumor cells were stained with antibodies to human IL-15 for 30 min. For the cell conjugation assay, 1.5 × 10^5^ purified NK cells were stained with CD56 antibody, placed in culture medium with 1.5 × 10^5^ CFSE-labeled CD19^+^ tumor cells, and treated with 161519 TriKE (10 μg/mL) or phosphate-buffered saline (PBS). Tumor cells were incubated for different time intervals at 37 °C, and then fixed in 1% paraformaldehyde. For the proliferation assay, PBMCs were co-cultured with or without Daudi cells at an effector cell/target cell (E:T) ratio of 5:1 in the presence of IL-15 (1 nM), 1619 BiKE (100 nM) or 161519 TriKE (100 nM). Three days later, cells were harvested and stained for cell-surface markers. After fixation and permeabilization, the cells were stained with antibody against Ki67.

### Flow cytometry

For flow cytometry, carboxyfluorescein succinimidyl ester (CFSE) was obtained from Invitrogen (Carlsbad, CA, USA). Phycoerythrin (PE)-conjugated antibodies to human IL-15 were obtained from R&D Systems (catalog number, 34559; Minneapolis, MN, USA). TNF-α (MAb11), TNF-related apoptosis-inducing ligand (TRAIL; RIK-2) and CD107a (H4A3) were from BD Biosciences (Franklin Lakes, NJ, USA). PerCP-Cy5.5-conjugated antibodies to human CD3 were from BioLegend (HIT3a; San Diego, CA, USA). Alexa Fluor 647-conjugated antibodies to human CD56 (B159) and Ki67 (B56) were purchased from BD Biosciences. Alexa Fluor 647-conjugated 161519 antibody was labeled by an Antibody Labeling Kit according to the manufacturer’s instructions (GeneCopoeia, Rockville, MD, USA).

For staining of TRAIL, CD107a, and intracellular cytokines, PBMCs were co-cultured with tumor cells in the presence of 161519 TriKE and monensin (2.5 μg/mL; eBioscience, San Diego, CA, USA) for 4 h. After incubation, tumor cells were stained for surface markers, fixed, and permeabilized with FoxP3 fixation buffer according to the manufacturer’s instructions (eBioscience). The fixed cells were then stained with antibodies to IFN-γ and TNF-α. All samples were acquired on an LSR-II or FACSCalibur flow cytometer (Becton Dickinson, Franklin Lakes, NJ, USA) and were analyzed using FlowJo (TreeStar, Ashland, OR, USA).

### Immunofluorescence assay

Purified NK cells and Raji cells were mixed with PBS or 161519 TriKE (10 μg/mL) and incubated for 30 min at 37 °C. The cells were then fixed in 1% paraformaldehyde and mounted on slides. Samples were blocked for 1 h at room temperature in 1% bovine serum albumin/PBS and stained with CD56 antibody (3576S; Cell Signaling Technology, Danvers, MA, USA) and CD19 antibody (ab134114; Abcam, Cambridge, UK) followed by Alexa Fluor Plus 647-conjugated goat anti-mouse antibody (A32728; Thermo Fisher Scientific, Waltham, MA, USA) and Alexa Fluor Plus 488-conjugated goat anti-rabbit antibody (A32731; Thermo Fisher Scientific). All slides were stained with 4′,6-diamidino-2-phenylindole (Thermo Fisher Scientific) for 4 min and mounted on coverslips in ProLong™ Gold anti-fade solution (Thermo Fisher Scientific). All procedures were conducted at room temperature. Slides were visualized using a LSM880 confocal laser scanning microscope (Zeiss, Oberkochen, Germany).

### Enzyme-linked immunosorbent assay (ELISA)

IFN-γ and TNF-α in cell supernatants were measured by ELISA kits according to the manufacturer’s instructions (Dakewe Biotech, Shenzhen, China).

### Cytotoxicity assay

Namalwa, Daudi, and MM.1S cells were labeled with CFSE, and incubated with purified human NK cells at indicated E:T ratios in the presence of 161519 TriKE, 1619 BiKE, or PBS for 4 h. For the spontaneous-death control, CFSE-labeled target cells were cultured alone under identical conditions. After 4 h, the wells were harvested and 7-aminoactinomycin D (7AAD) was added before analyses. Samples were mixed thoroughly and analyzed by flow cytometry.

### *In vivo* mouse models and bioluminescence imaging

#### Antitumor activity of 161519 TriKE

NOG mice were injected intravenously (i.v.) with luciferase-expressing Namalwa (Namalwa-luc) tumor cells (1 × 10^6^) on day 0 followed by injection i.v. of human PBMCs (1 × 10^7^) on day 5. Tumor-bearing mice were injected intraperitoneally (i.p.) with 161519 TriKE or 1619 BiKE (50 μg) every 2 days for a total of 10 doses beginning on day 6.

#### Antitumor activity of 161519 TriKE combined with IL-2

NOG mice were injected i.v. with Namalwa-luc tumor cells (1 × 10^6^) on day 0 followed by injection i.v. of human PBMCs (1 × 10^7^) on day 4. Tumor-bearing mice were injected i.p. with 161519 TriKE or 1619 BiKE (50 μg) combined with IL-12 (50,000 units; Jiangsu Kingsley Pharmaceuticals, Nanjing, China) every 2 days for a total of 10 doses beginning on day 4.

#### Bioluminescence imaging

Mice were injected i.p. with d-luciferin (15 mg/mL; Gold Biotechnology, St. Louis, MO, USA) at 150 mg/kg bodyweight 15 min before imaging 14 days after tumor challenge. Mice were placed into an *in vivo* imaging system (Caliper Life Sciences, Waltham, MA, USA) when fully anesthetized by isoflurane. Luciferase expression was imaged using Spectral In Vivo (PerkinElmer, Waltham, MA, USA) and calculated using Living Image (PerkinElmer).

### Statistical analysis

Comparisons between 2 groups were analyzed using the unpaired Student’s *t*-test. Comparisons between ≥ 3 groups were analyzed using 2-way analysis of variance. Kaplan-Meier analyses and Mantel-Cox tests were used to analyze mouse survival. Results are expressed as the mean ± SEM. *P* < 0.05 was assumed to be significant in all analyses.

## Results

### Construction and binding specificity of 161519 TriKE

**Figure 1:**
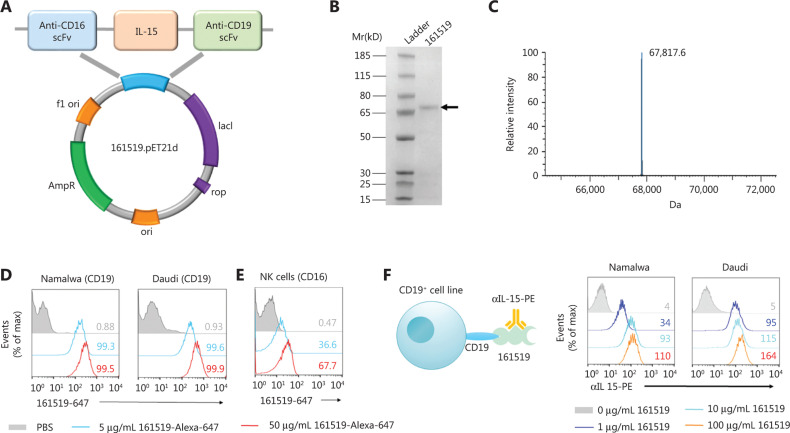
Construction and binding specificity of 161519 trispecific killer engager (TriKE). (A) Schematic representation of 161519 TriKE. The genes of an anti-CD16 scFv, a N72D-mutated human IL-15 flanked with 2 linkers, and an anti-CD19 scFv were inserted into a pET-21d expression vector. (B) The molecular weight and purity of 161519 TriKE were determined by SDS-PAGE. (C) ESI-TOF-MS of 161519 TriKE. (D) Binding specificity of 161519 TriKE with CD19. The 161519 TriKE was labeled with Alexa Fluor 647 fluorescein and incubated together with a CD19^+^ cell line (Namalwa or Daudi) at the concentrations indicated, followed by flow cytometry. The numbers in the graph represent the mean positive proportion. (E) Binding specificity of 161519 with CD16. The 161519 TriKE was labeled with Alexa Fluor 647 fluorescein and incubated with CD16^+^ cells (purified human NK cells) at the concentrations indicated, followed by flow cytometry. The numbers in the graph represent the mean positive proportion. (F) Binding specificity of 161519 TriKE with anti-IL15. Namalwa or Daudi cells were incubated with 161519 TriKE at various concentrations, and 161519-binding cells were detected with PE-conjugated human IL-15 antibody by flow cytometry after removing unbound 161519 TriKE. The numbers in the graph represent mean fluorescence intensity (MFI).

We aimed to improve the function of NK cells, so we inserted human IL-15 into 1619 BiKE to construct a new 161519 TriKE, which contained an anti-CD16 scFv, a human IL-15 flanked with 2 linkers, and an anti-CD19 scFv (**Figure 1A**). Expression of the 161519 gene was induced by the addition of IPTG into the bacterial-expression system, and 161519 protein was refolded from bacterial inclusion bodies (**Supplementary Figure S1A**). The purity of the refolded product was > 90% as determined by SDS-PAGE (**Figure 1B**). The precise molecular mass of 161519 TriKE was determined to be 67.8 kD using LC-ESI-TOF-MS (**Figure 1C**).

To ascertain the binding ability of 161519 TriKE, Namalwa and Daudi cells were incubated with Alexa Fluor 647-labeled 161519 TriKE in the presence of PBS, 161519 TriKE (5 μg/mL), or 161519 TriKE (50 μg/mL). Addition of Alexa Fluor 647-labeled 161519 TriKE increased the proportion of Alexa Fluor 647-positive Namalwa and Daudi cells (both: ˜100%), indicating that 161519 TriKE could bind to these CD19^+^ target cells through a specific interaction with CD19 (**Figure 1D**). In addition, incubation of purified human NK cells with Alexa Fluor 647-labeled 161519 TriKE increased the proportion of Alexa Fluor 647-positive human NK cells. A total of 67.7% of purified human NK cells were positive for the presence of 161519 protein, indicating specific binding between 161519 TriKE and human NK cells through the interaction with CD16 (**Figure 1E**). In contrast, specific binding was not observed between 161519 TriKE and T cells (CD16^−^) or MM.1S cells (CD19^−^) when they were incubated together (**Supplementary Figure S1B and S1C**). Furthermore, we incubated Namalwa and Daudi cells with 161519 TriKE (0, 1, 10, or 100 μg/mL) and then measured IL-15 expression on the surface of these target cells by PE-labeled anti-IL-15 monoclonal antibody (mAb), showing that anti-IL-15 mAb bound to IL-15 fragments of 161519 TriKE in a dose-dependent manner (**Figure 1F**).

### The 161519 TriKE significantly improves the interaction between NK cells and CD19^+^ tumor cells

**Figure 2:**
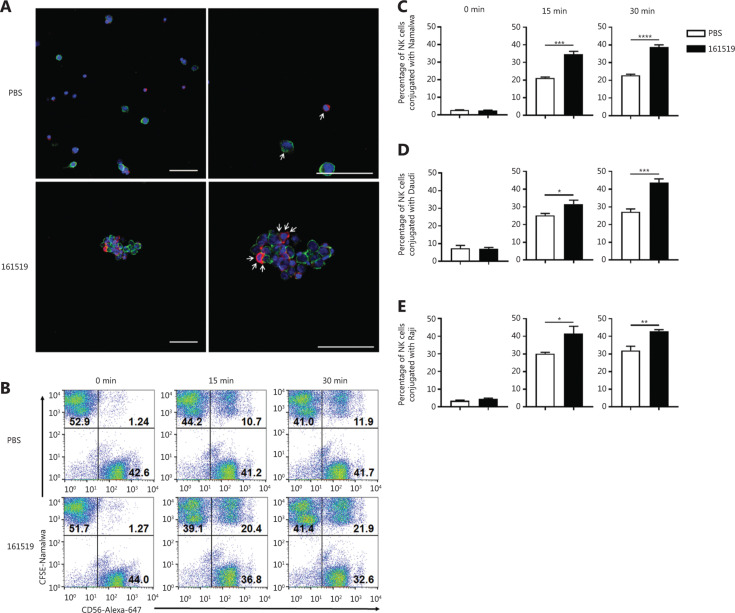
Conjugation of natural killer (NK) cells with CD19-expressing cells in the presence of 161519 trispecific killer engager (TriKE). (A) Joining of NK cells and tumor cells in the presence of 161519 TriKE. Purified NK cells and Raji cells were mixed with phosphate-buffered saline (PBS) or 161519 TriKE (10 μg/mL) and incubated at 37 °C, followed by staining of CD56 (red), CD19 (green), and DAPI (blue). Accumulation of cells was observed by confocal immunofluorescence microscopy in the presence of 161519 TriKE, scale bar = 50 μm. (B-D) Purified NK cells (CD56^+^) and CFSE-labeled CD19^+^ (Namalwa, Daudi, or Raji) cells were mixed with PBS or 161519 TriKE (10 μg/mL) and incubated at 37 °C for 0, 15, or 30 min. Cells were stained with 647-CD56 and analyzed by flow cytometry. The conjugation ratio was calculated by the proportion of FITC/Alexa-647 double-positive events within Alexa-647-positive events. (B) The representative percentage of NK cells binding to Namalwa cells is shown. The cumulative percentage of NK cells conjugated with (C) Namalwa, (D) Daudi, and (E) Raji cells is shown. Data are representative of 3 independent experiments and were analyzed by the Student’s *t*-test. *P < 0.05; **P < 0.01; ***P < 0.001; ****P < 0.0001.

To verify that 161519 TriKE promoted binding of NK cells to CD19^+^ tumor cells, purified NK and Raji cells were co-cultured in the presence of PBS or 161519 TriKE. Close interaction of NK cells (red) and Raji cells (green) was observed in the presence of 161519 TriKE under a confocal immunofluorescence microscope (**Figure 2A**). In addition, 161519 TriKE was added to the co-culture system of purified NK cells stained with Alexa Fluor 647-CD56 mAb and CFSE-labeled Namalwa cells for 0, 15, or 30 min. Addition of 161519 TriKE into the co-culture system for 15 and 30 min significantly increased the binding between NK cells and Namalwa cells, when compared with that of PBS alone (*P* < 0.001; *P* < 0.0001, respectively) (**Figure 2B and 2C**). Similar results were observed when using Daudi and Raji cells as target cells; addition of 161519 TriKE significantly increased the binding between NK cells and these target cells, when compared with that of PBS alone (**Figure 2D and 2E**). Furthermore, 161519 TriKE showed better performance in the conjugation of NK cells and CD19^+^ tumor cells (Namalwa and Daudi cells), when compared with that using rituximab (**Supplementary Figure S2**). These results suggested that NK cells conjugated more effectively with CD19^+^ tumor cells after treatment with 161519 TriKE.

### The 161519 TriKE significantly enhances the activation, proliferation, and cytokine secretion of NK cells

**Figure 3:**
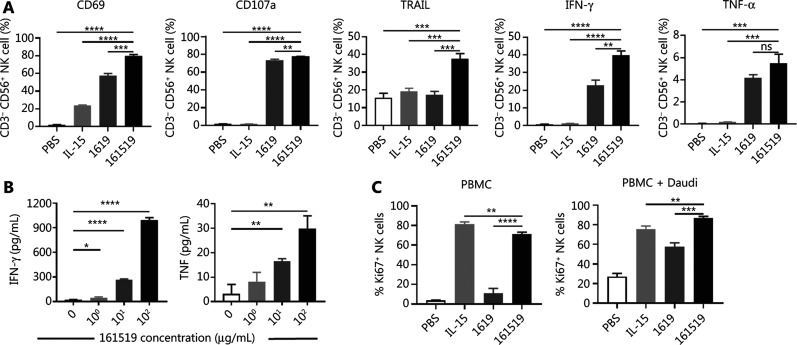
Activation and proliferation of natural killer (NK) cells by CD19-expressing cells in the presence of 161519 trispecific killer engager (TriKE). (A) Peripheral blood mononuclear cell (PBMCs) were co-cultured with Daudi cells at an E:T ratio of 5:1 in the presence of PBS, IL-15 (1 nM), 1619 BiKE (100 nM) or 161519 TriKE (100 nM) for 4 h, and CD3^−^CD56^+^ NK cells were assessed for expression of CD69, CD107a, TRAIL, IFN-γ and TNF-α by flow cytometry. (B) Purified NK cells were co-cultured with Namalwa cells at an E:T cell ratio of 1:1 in the presence of 161519 TriKE at the concentrations indicated for 24 h, and supernatants were assessed for levels of secreted IFN-γ and TNF-α using ELISAs. (C) PBMCs were incubated with IL-15 (1 nM), 1619 BiKE (100 nM) or 161519 TriKE (100 nM) in the presence or absence of Daudi cells for 3 days, and CD3^−^ CD56^+^ NK cells were assessed for Ki67 by flow cytometry. Data are representative of 3 independent experiments and analyzed by the Student’s *t*-test. *P < 0.05; **P < 0.01; ***P < 0.001; ****P < 0.0001.

To investigate the effect of 161519 TriKE on the activation and function of NK cells, PBMCs were incubated with Daudi cells (PBS), or in combination with IL-15, 1619 BiKE, or 161519 TriKE. Treatment with 161519 TriKE significantly enhanced expression of CD69, CD107a, TRAIL, and IFN-γ in CD3^−^CD56^+^ NK cells compared with treatment with PBS, IL-15, or 1619 BiKE (**Figure 3A**). Treatment with 161519 TriKE significantly enhanced expression of TNF-α in NK cells compared with treatment with PBS or IL-15 (**Figure 3A**). In addition, dose-dependent upregulation of expression of IFN-γ, TNF-α, and TRAIL in NK cells was found after treatment with 161519 TriKE (**Supplementary Figure S3A**). Similar dose-dependent upregulation of expressions of IFN-γ, TNF-α, and TRAIL were observed when PBMCs were incubated with Namalwa cells in the presence of 161519 TriKE (**Supplementary Figure S3B**). Furthermore, cytokine secretion in supernatants was analyzed after co-culture of purified NK and Namalwa cells with or without 161519 TriKE. The 161519 TriKE significantly enhanced secretion of IFN-γ and TNF-α in the supernatant (*P* < 0.0001; *P* < 0.01) (**Figure 3B**). Co-culture of PBMCs with Daudi cells in the presence of 161519 TriKE shows a significantly higher proportion of Ki67^+^ NK cells compared with that seen upon treatment with IL-15 or 1619 BiKE (**Figure 3C**), which suggested that 161519 TriKE helped to sustain the proliferation of NK cells. Taken together, these results suggested that 161519 TriKE enhanced the activation, proliferation, and anti-tumor cytokine production of NK cells.

### The 161519 TriKE significantly enhances the cytotoxicity of NK cells against CD19^+^ tumor cells

**Figure 4:**
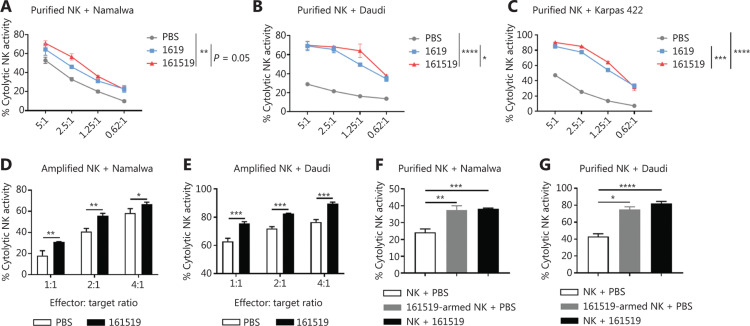
Cytotoxicity of natural killer (NK) cells against CD19^+^ tumor cells in the presence of 161519 trispecific killer engager (TriKE). CD19^+^ (A) Namalwa cells, (B) Daudi cells, or (C) Karpas 422 cells were labeled with CFDA-SE and incubated with purified human NK cells at an E:T ratio of 5:1, 2.5:1, 1.25:1 or 0.62:1 with phosphate-buffered saline (PBS), 1619 BiKE (15 nM), or 161519 TriKE (15 nM) for 4 h. Then, 7AAD was added and cytotoxicity (CFSE^+^ 7AAD^+^) was analyzed by flow cytometry. (D) Namalwa cells or (E) Daudi cells were labeled with CFDA-SE and incubated with NK cells amplified *in vitro* at an E:T ratio of 4:1, 2:1, or 1:1 with PBS or 161519 TriKE (10 μg/mL) for 4 h. Then, 7AAD was added and cytotoxicity (CFSE^+^ 7AAD^+^) was analyzed by flow cytometry. (F, G) Purified human NK cells were incubated with 161519 TriKE (10 μg/mL) for 30 min (161519-armed NK) followed by the removal of unbound antibody. Armed NK cells and untreated NK cells were incubated with CFSE-labeled (F) Namalwa cells or (G) Daudi cells, respectively, at an E:T ratio of 5:1 with PBS or 161519 TriKE (10 μg/mL) for 4 h. Then, 7AAD was added and cytotoxicity (CFSE^+^ 7AAD^+^) was analyzed by flow cytometry. Data are representative of 3 independent experiments and analyzed by Student’s *t*-test or 2-way analysis of variance. *P < 0.05; **P < 0.01; ***P < 0.001; ****P < 0.0001.

Anti-tumor cytotoxicity is one of the most important functions of NK cells. We aimed to analyze the anti-tumor cytotoxicity of NK cells under the influence of 161519 TriKE. Purified NK cells were co-cultured with Namalwa, Daudi, or Karpas 422 cells in the presence of PBS or 161519 TriKE for 4 h, followed by a cytotoxicity assay. The 161519 TriKE significantly improved the specific lytic ability of NK cells against CD19^+^ tumor cells (**Figure 4A–4C**). In contrast, use of 161519 TriKE in the co-culture of purified NK and CD19^−^ MM.1S cells showed no improvement in lysis of NK cells, which indicated the importance of CD19-specificity in this type of scenario (**Supplementary Figure S4**). Most of the products of human NK cells available in clinical settings are *in vitro* expanded human NK cells. Hence, we examined the effect of 161519 TriKE on the lysis of expanded human NK cells. Use of 161519 TriKE significantly improved the lytic activity of amplified NK cells against Namalwa and Daudi cells, when compared with that using PBS alone at all 3 E:T ratios tested (**Figure 4D, 4E**). Notably, purified NK cells armed with 161519 TriKE for 30 min (161519-armed NK) showed stronger lysis against Namalwa and Daudi cells without additional 161519 TriKE in the co-culture system (**Figure 4F, 4G**). Hence, these 161519-armed NK cells were redirected with strong targeting efficiency to CD19^+^ tumor cells, which could be an alternative approach for using NK cells in clinical cellular therapy.

### The 161519 TriKE delays tumor growth significantly and prolongs survival in an immune-reconstituted xenograft model in mice

**Figure 5:**
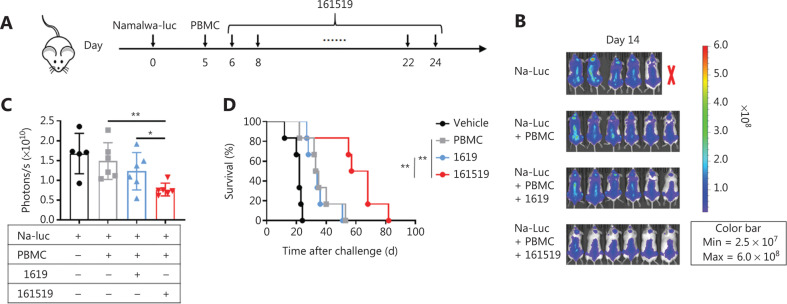
Antitumor activity of 161519 trispecific killer engager (TriKE) in human peripheral blood mononuclear cell (PBMC)-reconstituted xenograft mice. (A) Experimental protocol for the lymphoma model used in (B–D): NOG mice (*n* = 6/group) were injected intravenously (i.v.) with Namalwa-luc tumor cells (1 × 10^6^) on day 0 followed by intravenous injection of human PBMCs (1 × 10^7^) on day 5. Tumor-bearing mice, randomized on day 6, were injected intraperitoneally with 161519 TriKE or 1619 BiKE (50 μg) every 2 days for a total of 10 doses beginning on day 6. (B) *In vivo* bioluminescence imaging in mice 14 days after the tumor challenge described in (A). (C) Quantification of luminescence in mice from the 4 treatment groups described in (A) on day 14. (D) Survival of mice from different treatment groups as described in (A). Each symbol (C, D) represents an individual mouse. Data were analyzed by Student’s *t*-test (C) and the Mantel-Cox test (D). *P < 0.05; **P < 0.01.

We aimed to assess if 161519 TriKE improved the antitumor function of NK cells *in vivo*. Hence, we established a xenograft model involving constituted human PBMCs using NOG mice. These mice were loaded with Namalwa lymphoma cells and treated with 161519 TriKE (**Figure 5A**). Tumor growth was significantly inhibited after treatment with 161519 TriKE. Moreover, 161519 TriKE treatment showed a stronger effect at delaying tumor growth, when compared with that observed with 1619 BiKE treatment (*P* < 0.05) (**Figure 5B, 5C**). Consistent with the results of tumor growth, xenografted mice treated with 161519 TriKE showed significantly improved survival compared with that of the control and 1619-BiKE treatment groups (*P* < 0.01 for both) (**Figure 5D**), which suggested the potential value of 161519 TriKE in treating CD19^+^ tumors *in vivo*.

### The 161519 TriKE combined with IL-2 significantly delays tumor growth and prolongs survival in an immune-reconstituted xenograft model in mice

**Figure 6:**
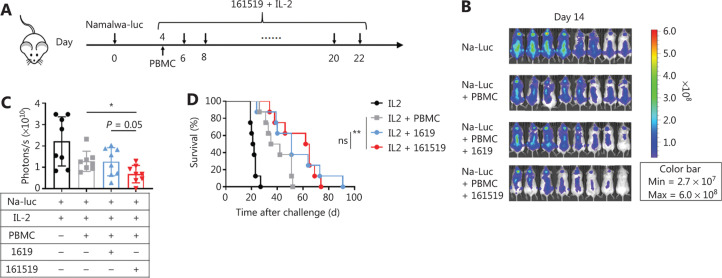
Antitumor activity of 161519 trispecific killer engager (TriKE) combined with IL-2 in human peripheral blood mononuclear cell (PBMC)-reconstituted xenograft mice. (A) Experimental protocol for the lymphoma model used in (B-D): NOG mice (*n* = 8/group) were injected intravenously (i.v.) with Namalwa-luc tumor cells (1 × 10^6^) on day 0, followed by intravenous injection of human PBMCs (1 × 10^7^) on day 4. Tumor-bearing mice, randomized 4 h later, were injected intraperitoneally with 161519 TriKE or 1619 BiKE (50 μg) combined with human IL-2 (50,000 units) every 2 days for a total of 10 doses beginning on day 4. (B) *In vivo* bioluminescence imaging in mice 14 days after the tumor challenge described in (A). (C) Quantification of luminescence in mice from the 4 treatment groups described in (A) on day 14. (D) Survival of mice from different treatment groups as described in (A). Each symbol (C, D) represents an individual mouse. Data were analyzed by Student’s *t*-test (C) and the Mantel-Cox test (D). *P < 0.05; **P < 0.01.

We aimed to determine the necessity of IL-2 therapy during treatment with 161519 TriKE *in vivo*. Hence, we further compared the use of 161519 TriKE and 1619 BiKE under the influence of IL-2 regimens using a xenograft murine model loaded with Namalwa lymphoma cells (**Figure 6A**). As expected, 161519 TriKE combined with IL-2 treatment significantly delayed tumor growth and prolonged the survival of xenografted mice compared with that observed in controls (**Figure 6B–6D**). However, significant differences were not observed between the survivals of mice treated with 161519 TriKE combined with IL-2 and those treated with 1619 BiKE combined with IL-2. These results suggested that IL-2 probably had a more important role when used in combination with 1619 BiKE than in combination with 161519 TriKE. Combined use of IL-2 with 1619 BiKE reduced the significant differences in the tumor growth and survival between the 2 treatment groups (1619 BiKE *vs*. 161519 TriKE). Taken together, these data suggested that 161519 TriKE delayed tumor growth, prolonged overall survival of xenografted mice, and showed a stronger effect for treating tumors compared with that using 1619 BiKE.

## Discussion

Cold tumors are immunologically ignorant and express minimal or no MHC class-I molecules on the cell surface^7^. They are barely recognized by CD8^+^ T cells due to the missing/impaired expression of MHC class-I molecules, but they can be detected readily and targeted by NK cells^8,9,23^. The 161519 TriKE constructed in our studies was a trispecific protein developed for the treatment of B-cell malignancies (CD19-positive cancers) and was designed to redirect NK cells *via* their activating receptor CD16 to kill target cells expressing CD19. An IL-15 signal was introduced into the platform to sustain the proliferation, homeostasis, activation, and survival of NK cells^24^. The 161519 TriKE bound specifically to CD16, CD19, and anti-IL-15, which is consistent with a report by Felices et al.^20^. The 161519 TriKE improved the interaction between NK cells and CD19^+^ tumor cells and eliminated pathogenic CD19^+^ targets effectively *in vitro*. The overall structures of 161519 TriKE in our study and the one designed previously^20^ were identical. However, the anti-CD16 and anti-CD19 antibody sequences^16,23^ used in our study were different from the ones used previously^25,26^. In addition, our study used *in vivo* models to show that 161519 TriKE promoted the proliferation and survival of human NK cells and mediated stronger anti-tumor responses compared with that by 1619 BiKE.

Treatment with 161519 TriKE in the presence of CD19^+^ targets resulted in enhanced expression of CD69, CD107a, TRAIL, IFN-γ, and TNF-α in NK cells. Felices et al.^20^ reported enhanced degranulation (CD107a expression) of NK cells after treatment with 161519 TriKE in the presence of CD19^+^ targets. Our study provided additional mechanistic details involving TRAIL, which supported the enhancement of cytotoxicity against CD19^+^ targets in the presence of 161519 TriKE. TRAIL (also known as Apo2 ligand) is a type-II transmembrane protein belonging to the TNF superfamily. TRAIL is often involved in the toxic activity of activated NK cells against TRAIL-sensitive tumor cells *in vitro*^27^. Furthermore, IFN-γ regulates TRAIL expression on effector cells and sensitizes tumor cells to TRAIL-mediated cytotoxicity^28^. Dose-dependent upregulation of expression of IFN-γ and TRAIL in NK cells by 161519 TriKE suggested that 161519-mediated cytotoxicity might also be dependent upon TRAIL expression.

T cell-dependent BsAbs have shown substantial clinical efficacy by activating endogenous T cells. The ability of T cells to secrete cytokines is used as a marker for T-cell activity and their cytolytic potential^29^. The CD19-targeting BiTE blinatumomab has achieved substantial clinical efficacy against cancer cells. Continuous intravenous infusion of blinatumomab has been shown to result in CR or CRh in 43% of patients with R/R B-precursor^4,5^. Despite these promising outcomes, uncontrolled systemic cytokine release and neurological toxicity hinder practical use of T cell-dependent therapies^2,30^. T cell-produced TNF-α causes monocyte activation, which could lead to systemic production of toxic cytokines^29^. Moreover, IL-6 is crucial for treatment of fever induced by forskolin, TNF-α, or IL-1^31^. Also, increased expression of IL-6 is detected frequently in CRS, and has been suggested to have a central role in the clinical progression of CRS^32^. Recently, Liu et al.^33^ showed that, unlike chimeric antigen receptor (CAR)-T cells (which induce substantial toxic effects), CAR-NK cells targeting CD19^+^ cancers were not associated with the development of CRS, neurotoxicity, or graft-versus-host disease, and an increase in expression of inflammatory cytokines (including IL-6) was not observed. We showed that the overall level of TNF-α production in NK cells was very low after treatment with 161519 TriKE, which may be one of the reasons why TriKE exerted a minimal effect upon CRS. NK cells activated by 161519 TriKE might therefore produce less therapeutic-based toxicity compared with that of T cells activated by BiTE, which suggests a promising therapy to maximize clinical success.

Clinical trials have shown that infusions of NK cells are well-tolerated without causing CRS^34^. However, the clinical efficacy of *in vitro* expanded primary NK cells or NK cell lines is limited^35^. We showed that *in vitro* expanded NK cells exhibited enhanced killing ability against tumor cells. Large-scale, clinical-grade expansion methods for NK cells have been well established. Our findings indicated enhanced tumor control when *in vitro* expanded NK cells were used in combination with 161519 TriKE, which could provide new and effective therapeutic combinations. Importantly, purified NK cells armed with 161519 TriKE showed stronger lysis against CD19^+^ tumor cells, when compared with that of unarmed NK cells, indicating stronger targeting efficiency of 161519-armed NK cells and their potential for use in clinical cellular therapy.

Investigators have reported the generation of BsAbs consisting of mAbs and cytokines. Human IL-2 linked to mAbs has been used for phase-I studies for the therapy of melanoma and prostate cancer^36,37^. IL-15 shares common receptor components with the IL-2 receptor, but is less toxic and also crucial for the development of NK cells^38^. The 1633 BiKE fused with a human IL-15 crosslinker shows the enhanced ability of NK-cell proliferation and *in vivo* superior antitumor activity against CD33^+^ targets, when compared with that using 1633 BiKE^17^. The 161533 TriKE restores tumor-induced dysfunction and repression of NK cells^17,18^. In addition, 161519 TriKE induces NK-cell proliferation, rescues the inflammatory function of NK cells obtained from CLL patients, and induces better directed killing of CLL targets *in vitro*^20^. Consistent with those findings, we also reported that the *in vitro* cytotoxicity of NK cells treated with 161519 TriKE was stronger compared with that of controls or those treated with 1619 BiKE. Furthermore, 161519 TriKE could induce proliferation. In the xenograft model involving human PBMCs, longer lasting anti-tumor activity was observed in tumor-bearing mice treated with 161519 TriKE compared with that in mice treated with 1619 BiKE. Furthermore, IL-2 probably has a more important role when used in combination with 1619 BiKE, when compared with 161519 TriKE, because combined use of IL-2 with 1619 BiKE reduced the significant differences in the tumor growth and survival between the two treatment groups (1619 BiKE *vs*. 161519 TriKE).

## Conclusions

NK cells are powerful tools against cancer, and they can be efficacious in cases when T cells fail^13,39^. Moreover, anti-tumor products involving NK cells show fewer adverse effects than those involving T cells^16,33^. Newly generated 161519 TriKE in our study enhanced the activation, proliferation, and function of NK cells and showed effective anti-tumor activity *in vivo*. It can therefore be used as anti-tumor treatment or to arm NK cells in advance in NK cellular therapy, thereby providing a promising way to treat CD19-positive cancers.
