# Supplementary material for: Trispecific killer engager 161519 enhances natural killer cell function and provides anti-tumor activity against CD19-positive cancers
Source: Cancer Biol Med. 2020 Dec 15;17(4):1026–38. doi: 10.20892/j.issn.2095-3941.2020.0399 (PMC7721099; doi:10.20892/j.issn.2095-3941.2020.0399)
Supplement: Supplementary file 1 [file cbm-17-1026-s001.pdf]

Supplementary materials

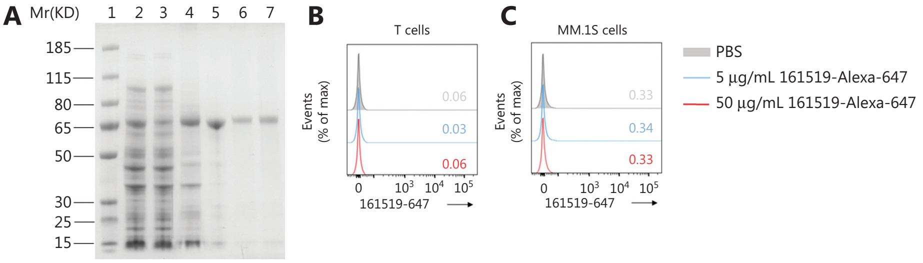

Figure S1 Expression and binding specificity of 161519 trispecific killer engager (TriKE). (A) Bacteria were induced by addition of isopropyl-β-D-thiogalactopyranoside (IPTG). and inclusion bodies were refolded using a sodium N-lauryl-sarcosine air-oxidation method. Column 1: molecular weight standards; column 2: induced bacterial lysate by IPTG; column 3: supernatant after sonication and centrifugation; column 4: insoluble substance after sonication and centrifugation; column 5: insoluble substance after inclusion bodies were washed 4 times; column 6: TriKE solution after incubation with refolding buffer; and column 7: refolded TriKE after buffer was exchanged. (B, C) 161519 TriKE was labeled with Alexa Fluor 647 fluorescein and incubated together with (B) human T cells or (C) MM.1S cells at the concentrations indicated, followed by flow cytometry. The numbers in the graph represent the mean positive proportions.

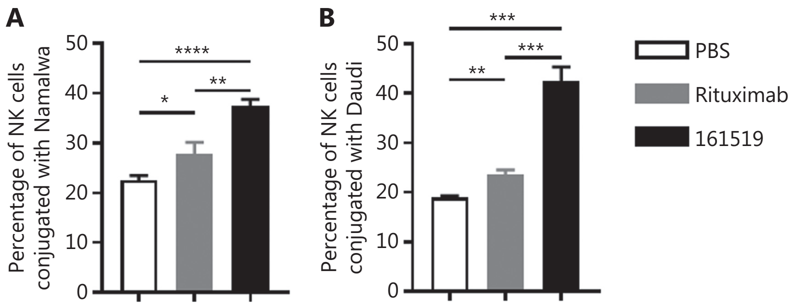

Figure S2 Comparison between 161519 trispecific killer engager (TriKE) and rituximab in the conjugation of natural killer (NK) cells and tumor cells. Purified NK cells ($CD56^+$) and CFSE-labeled (A) Namalwa cells and (B) Daudi cells were mixed with phosphate-buffered saline, rituximab (100 nM), or 161519 TriKE (100 nM) and incubated for 30 min at 37 °C. The cells were stained with 647-CD56 and analyzed by flow cytometry. The conjugation ratio was calculated by the proportion of FITC/Alexa-647 double-positive events within Alexa-647-positive events. Data are representative of 3 independent experiments and analyzed using the Student's *t*-test. *P < 0.05; **P < 0.01; ***P < 0.001; ****P < 0.0001.

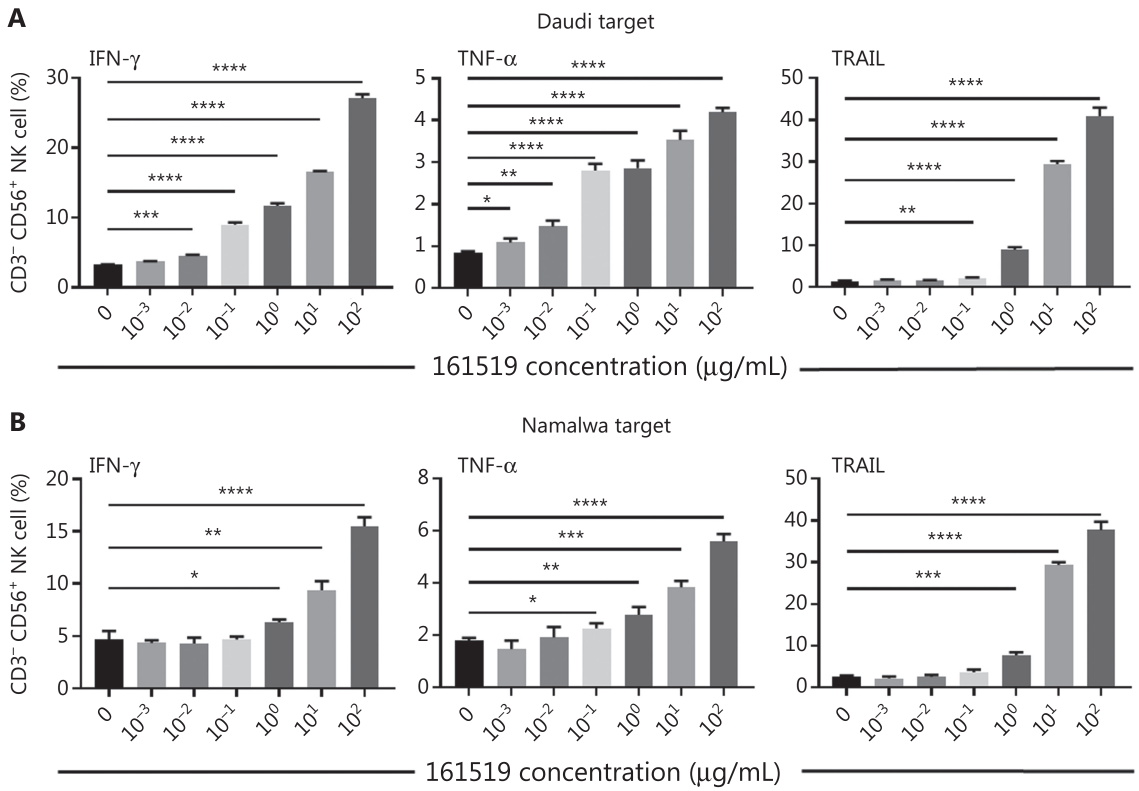

Figure S3 Dose-dependent activation of natural killer (NK) cells by CD19-expressing target cells in the presence of 161519 trispecific killer engager (TriKE). Peripheral blood mononuclear cells were co-cultured with (A) Daudi cells and (B) Nawalma cells at an E:T ratio of 10:1 in the presence of 161519 TriKE at the concentrations indicated for 4 h, and $CD3^-$ $CD56^+$ NK cells were assessed for production of IFN-γ, TNF-a, and TRAIL by flow cytometry. Data are representative of 3 independent experiments and analyzed using Student's *t*-test. *$P < 0.05$; **$P < 0.01$; ***$P < 0.001$; ****$P < 0.0001$.

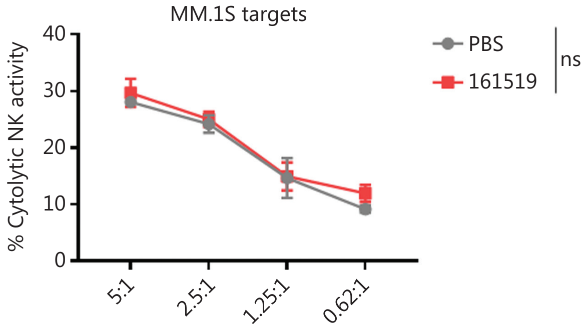

Figure S4 Cytotoxicity of natural killer (NK) cells against $CD19^-$ tumor cells in the presence of 161519 trispecific killer engager. $CD19^-$ MM.1S cells were labeled with CFDA-SE and incubated with purified human NK cells at indicated E:T ratios with phosphate-buffered saline or 161519 TriKE (10 μg/mL) for 4 h. Then, 7AAD was added and the cytotoxicity ($CFSE^+$ $7AAD^+$) was analyzed using flow cytometry. Data are representative of 3 independent experiments and analyzed using 2-way analysis of variance.
